# LOXL1-AS1 contributes to the proliferation and migration of laryngocarcinoma cells through miR-589-5p/TRAF6 axis

**DOI:** 10.1186/s12935-020-01565-5

**Published:** 2020-10-13

**Authors:** Guijun He, Wenfeng Yao, Liang Li, Yang Wu, Guojian Feng, Li Chen

**Affiliations:** 1 Department of Otolaryngology and Head and Neck Surgery, Lianyungang Second People’s Hospital, Lianyungang, 222023 Jiangsu China; 2 Department of Otolaryngology, The First People’s Hospital of Xinxiang City, Xinxiang, 453000 Henan China; 3 Department of Otorhinolaryngology, Zaozhuang Municipal Hospital, No. 41, Longtou Middle Road, Shizhong District, Zaozhuang, 277100 Shandong China

**Keywords:** Laryngocarcinoma, LOXL1-AS1, miR-589-5p, TRAF6

## Abstract

**Background:**

LOXL1-AS1 is a long non-coding RNA (lncRNA) that plays crucial roles in various cancers. However, the functional role of LOXL1-AS1 in laryngocarcinoma remains unclear. Thus we planned to probe into the function and underlying mechanism of LOXL1-AS1 in laryngocarcinoma.

**Methods:**

Gene expression was evaluated in laryngocarcinoma cells using RT-qPCR. The ability of cell proliferation and migration was assessed by CCK8, colony formation, wound healing and transwell assays. The interaction among LOXL1-AS1, miR-589-5p and TRAF6 was detected by Ago2-RIP, RNA pull down and luciferase reporter assays.

**Results:**

LOXL1-AS1 was overexpressed in laryngocarcinoma cells. Silencing of LOXL1-AS1 suppressed cell proliferation, migration and EMT in laryngocarcinoma. Moreover, miR-589-5p, the downstream of LOXL1-AS1, directly targeted TRAF6 in laryngocarcinoma. Importantly, LOXL1-AS1 augmented TRAF6 expression in laryngocarcinoma cells by sequestering miR-589-5p. Besides, miR-589-5p worked as a tumor-inhibitor while TRAF6 functioned as a tumor-facilitator in laryngocarcinoma. Of note, rescue experiments both in vitro and in vivo validated that LOXL1-AS1 aggravated the malignancy in laryngocarcinoma by targeting miR-589-5p/TRAF6 pathway.

**Conclusions:**

LOXL1-AS1 promotes the proliferation and migration of laryngocarcinoma cells through absorbing miR-589-5p to upregulate TRAF6 expression.

## Background

Laryngocarcinoma is a type of common malignant tumor of the upper respiratory tract [1]. Even though the current therapies containing surgical operation, radiotherapy and chemotherapy have an improved effect on laryngocarcinoma patients at early stage, laryngocarcinoma patients at advanced stage still suffer not optimistic prognosis [2]. Hence, uncovering the underlying molecular mechanism responsible for laryngocarcinoma carcinogenesis is essential for exploring effective treatment strategies.

As high-throughput sequencing technologies advanced, long non-coding RNAs (lncRNAs) are increasingly brought into focus. LncRNAs are longer than 200 nucleotides but cannot encode proteins [3]. Growing evidence has demonstrated that lncRNAs act as modulators to affect cellular activities in cancers, such as cell proliferation, migration, invasion and differentiation [4, 5]. For example, lncRNA CCAT2 facilitates cell growth and migration in hepatocellular carcinoma [6]. In recent years, LOXL1-AS1 is a new lncRNA that has been elucidated to play a vital part in sundry cancer types, like breast cancer [7], glioblastoma [8], prostate cancer [9], and cholangiocarcinoma [10]. Nevertheless, its role in laryngocarcinoma keeps veiled. In this work, we concentrated on investigating the functional role of LOXL1-AS1 in laryngocarcinoma.

LncRNAs have been testified to regulate mRNAs through serving as a competing endogenous RNA (ceRNA) via sponging microRNAs (miRNAs) in diverse malignancies. For example, lncRNA CTC-497E21.4 modulates miR-22/NET1 pathway to accelerate gastric cancer progression [11]. Besides, LOXL1-AS1 has also been disclosed as a ceRNA in former reports. For instance, LOXL1-AS1 serves as a ceRNA of PIK3CA by absorbing miR-142-5p to aggravate gastric cancer progression [12]. LOXL1-AS1 works as a tumor-driver in non-small-cell lung cancer through its sponging role to miR-342-3p [13]. Also, LOXL1-AS1 accelerates the progression of endometrial cancer via modulating miR-28-5p/PAP1B signaling [14]. However, the mechanism whereby LOXL1-AS1 functions in laryngocarcinoma cells need to be clarified. Thus, we probed into whether LOXL1-AS1 affected laryngocarcinoma development via a ceRNA mechanism, and the downstream miRNA and mRNA were also explored here.

In short, our study was designed to elucidate the role of LOXL1-AS1 in laryngocarcinoma. Moreover, the potential regulatory mechanism was also concentrated on in the present study, which might offer novel ideas for treating patients with laryngocarcinoma.

## Methods

### Cell culture

The human normal pharyngeal epithelial cell line NP69 and laryngocarcinoma cells (Tu-177, M4E, SNU-899, SNU-46, and AMC-HN-8), all were commercially acquired from American Type Culture Collection (ATCC; Rockefeller, Maryland USA) and Shanghai Honsun Biological Technology (Shanghai, China). DMEM (Gibco, Grand Island, NY, USA) was employed culturing cells in 5% CO_2_ at 37 °C. 10% fetal bovine serum (FBS; Gibco) with 1% penicillin–streptomycin (HyClone, Logan, UT, USA) was used for supplementing medium.

### Reverse transcription quantitative PCR (RT-qPCR)

The isolation of total RNA was from cultured cells was achieved by the TRIzol Reagent (Invitrogen, Carlsbad CA, USA), followed by the RNA converting into cDNA by use of PrimeScript™ RT reagent kit (Takara, Otsu, Japan). Then, cDNA samples were subjected to qPCR with SYBR green Supermix (Thermo Fisher, Waltham, MA, USA). Relative expression was determined by the comparative change-in-cycle (ΔΔCt) method, with GAPDH or U6 as the reference.

### Cell transfection

ShRNAs specific to LOXL1-AS1 and TRAF6, as well as the nonspecific control (sh/NC), were synthesized by GenePharma (Shanghai, China). Then, pcDNA3.1 vector (Invitrogen) covering full-length sequence of LOXL1-AS1 cDNA was applied for overexpressing LOXL1-AS1, with the empty vector as negative control (NC). Besides, miR-589-5p mimics, miR-589-5p inhibitor and corresponding NCs were constructed by Ribobio (Guangzhou, China). They were appropriately transfected into Tu-177 and M4E cells for 48 h by using Lipofectamine 2000 (Invitrogen). Each procedure was run in triplicate.

### Cell counting kit-8 (CCK-8) assay

The transfected laryngocarcinoma cells (5000 in each well) were planed into 96-well plates. After incubation for indicated times, each well was added with 10 μL of CCK-8 solution (Dojindo, Kumamoto, Japan) for treating cells for 2 h, as instructed by provider. Finally, the optical density (OD) value was examined at 450 nm using spectrophotometer (Thermo Fisher). Each procedure was run in triplicate.

### Colony formation assay

The transfected laryngocarcinoma cells were prepared in 6-well plates (1000 cells/well) for 14-day cell culture purposes. Then, cells were processed with 4% paraformaldehyde (PFA) for 30 min of fixing, and 0.5% crystal violet solution for 5 min of staining. The visible colonies were counted manually. Each procedure was run in triplicate.

### Wound healing assay

1 × 10^6^ laryngocarcinoma cells were seeded into 6-well plates until that cells were adhered to the plates. The artificial scratch was made by 200-μL pipette tip. At 0 and 24 h after culture in serum-free medium, the wound healing was observed and the images were acquired. Then, relative wound width was calculated using the relative value of wound widths at 24 h to that at 0 h. Each procedure was run in triplicate.

### Transwell migration assay

2 × 10^4^ transfected laryngocarcinoma cells in medium without serum were supplemented into the upper chamber of 24-well-Transwell insert (8-mm pore size; Corning Incorporated, Corning, NY, USA). The lower chamber was filled with the medium containing 10% FBS. Following 24 h of incubation, cells migrating to the bottom were fixated by 4% PFA. Cells in five random fields were counted with the aid of optical microscope (Olympus, Tokyo, Japan) after staining via crystal violet. Each procedure was run in triplicate.

### Immunofluorescence staining (IF)

2.5 × 10^4^ transfected laryngocarcinoma cells were seeded into each well of 6-well plates with glass coverslips. 24 h later, cells on coverslips were fixed for 1 h by 4% PFA at 25 °C, followed by 5 min of permeabilization by 0.1% Triton X-100. Then, cells were subjected to 30 min of blocking by 5% BSA and incubation with primary antibodies against E-cadherin (Cat# 14472S; Cell Signaling Technology, Danvers, MA, USA) and N-cadherin (Cat # 13116S; Cell Signaling Technology) all night at 4 °C. Following probing with secondary antibodies for 1 h at 37 °C, cells were observed by fluorescence microscope (Olympus). Each procedure was run in triplicate.

### Nuclear separation

Nuclear separation assay of laryngocarcinoma cells was undertaken by using Cytoplasmic and Nuclear RNA Purification Kit (Norgen, Belmont, CA, USA). The fractionation buffer and disruption buffer were used to separate cell fractions as guided by the supplier. After centrifugation, LOXL1-AS1 content in different fractions was assessed by RT-qPCR, utilizing GAPDH and U6 as respective cytoplasmic and nuclear references. Each procedure was run in triplicate.

### FISH

The specific RNA probe to LOXL1-AS1 was constructed by Ribobio for conducting FISH assay. Cells were fixed for 20 min, digested for 10 min and then rinsed by PBS. Thereafter, cells were cultured with LOXL1-AS1-probe in hybridization buffer all night. At length, DAPI solution (Beyotime, Shanghai, China) was added before fluorescent detection using fluorescence microscope (Olympus). Each procedure was run in triplicate.

### RNA pull down assay

RNA pull down experiment was brought out in laryngocarcinoma cells applying Pierce Magnetic RNA–Protein Pull-Down Kit (Thermo Fisher). Following lysing cells with RIPA lysis buffer, the obtained extracts were used for mixing with the biotinylated probes for LOXL1-AS1 or miR-589-5p. 30 μl of magnetic beads was then added for 2 h of incubation. The finally acquired RNA was analyzed by RT-qPCR. Each procedure was run in triplicate.

### RNA immunoprecipitation (RIP)

RIP assay was implemented in laryngocarcinoma cells with Magna RIP™ RNA-Binding Protein Immunoprecipitation Kit (Millipore, Bedford, MA, USA), following the user guide. After lysing with RIP lysis buffer, cell lysates were processed with magnetic beads bound with anti-Ago2 or control IgG antibody (Millipore). Then, RNAs in the immunoprecipitates were subjected to RT-qPCR analysis. Each procedure was run in triplicate.

### Luciferase reporter assay

For this experiment, the full-length fragments of LOXL1-AS1 and TRAF6 3′UTR covering wild-type or mutant miR-589-5p interacting sites were cloned into pmirGLO dual-luciferase reporter vectors (Promega, Madison, WI, USA). After that, the constructs (pmirGLO/LOXL1-AS1-WT/Mut and pmirGLO/3′UTR-WT/Mut), were individually co-transfected into laryngocarcinoma cells for 48 h with indicated plasmids. In the end, the luciferase activities were monitored using Dual-luciferase reporter assay system (Promega). Each procedure was run in triplicate.

### In vivo xenograft assay

To perform the animal experiments, male nude BALB/C mice with the age of 6 weeks were provided by Beijing Vital River Laboratory Animal Technology (Beijing, China). 1 × 10^6^ laryngocarcinoma cells were inoculated at the back of nude mice, with the size of originated tumors monitored every 4-day. The cells injected into four groups of mice were transfected with sh/NC, sh/LOXL1-AS1#1, sh/LOXL1-AS1#1 + antagomir-589-5p, or sh/LOXL1-AS1#1 + antagomir-589-5p + sh/TRAF6. After inoculation for 28 days, the mice were sacrificed via cervical decapitation, followed by weigh assessment of the dissected tumors. This work was approved by the Animal Research Ethics Committee of Lianyungang Second People’s Hospital.

### Statistical analyses

The results of three independently performed experiments were displayed as mean ± standard deviation (SD). The statistical analyses, in form of one-way ANOVA or Student’s t-test, were conducted using GraphPad Prism 6.0 software (GraphPad Software, Inc., La Jolla, CA, USA). It was taken as significant when P-value < 0.05.

## Results

### LOXL1-AS1 facilitates the malignant behaviors of laryngocarcinoma cells

**Fig. 1:**
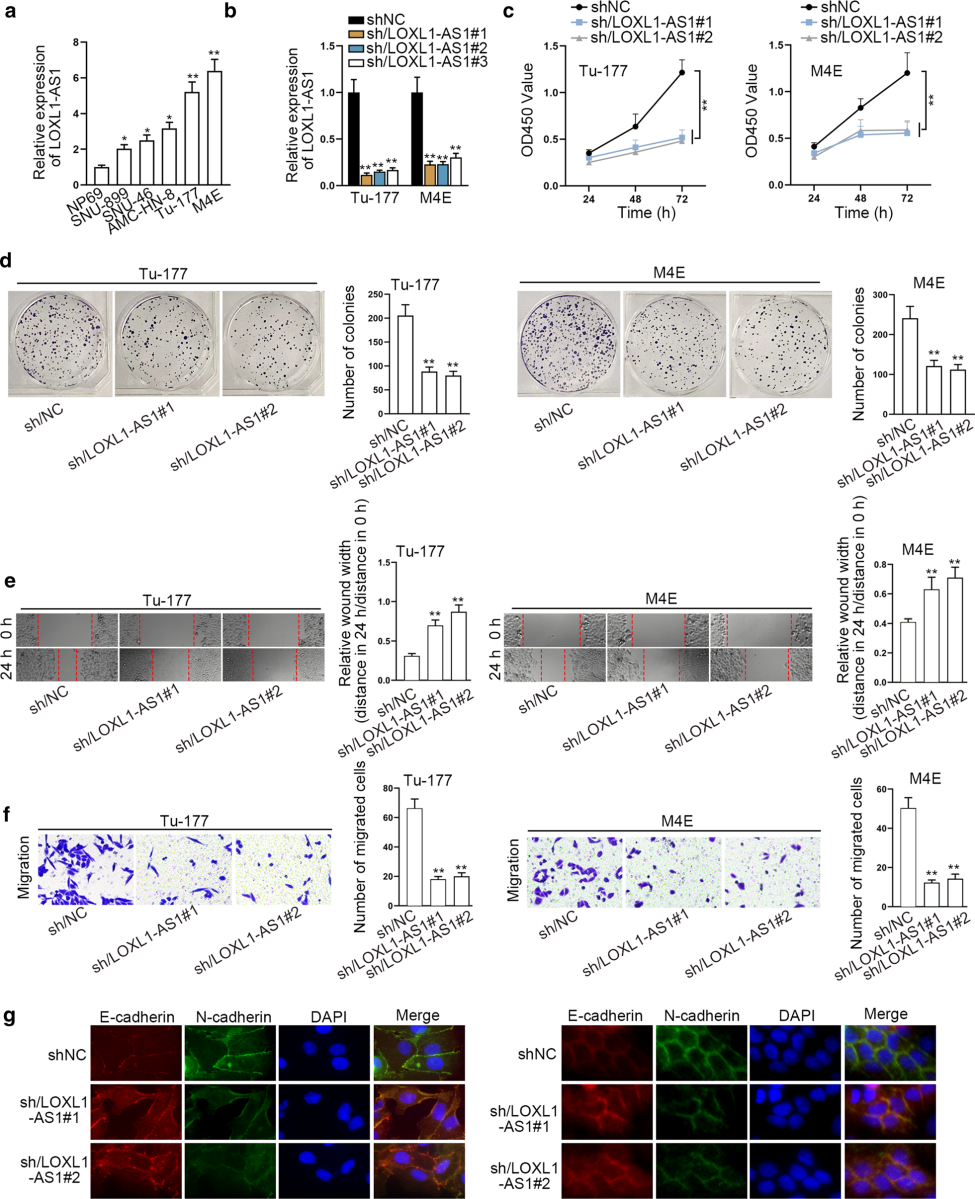
LOXL1-AS1 aggravates laryngocarcinoma cell proliferation, migration and EMT. **a** RT-qPCR analysis detected the overexpressed LOXL1-AS1in laryngocarcinoma cell lines (SNU-899, SNU-46, AMC-HN-8, Tu-177 and M4E) compared to normal pharyngal epithelial cell line (NP69). **b** The knockdown efficiency of LOXL1-AS1 was examined using RT-qPCR after transfecting three shRNAs targeting LOXL1-AS1 into Tu-177 and M4E cells. **c**, **d** CCK8 and colony formation assays detected the hindered cell proliferation in response to LOXL1-AS1 silencing. **e** Wound healing assay demonstrated the suppression on migration ability induced by LOXL1-AS1 silencing in Tu-177 and M4E cells. Relative wound width was calculated using the relative value of wound widths at 24 h to that at 0 h. **f** Transwell assay determined the hinder migration of Tu-177 and M4E cells with LOXL1-AS1 inhibition. G. IF examined the elevated expression of E-cadherin and the reduced level of N-cadherin in Tu-177 and M4E cells under LOXL1-AS1 inhibition. The standard deviation of control groups in **a** and **b** was calculated by 2^−ΔΔCt^ method as follow: ΔCt (Control) = ΔCt (target, Control) – ΔCt (reference, Control); mean ΔCt (Control) = {ΔCt−1 (Control) + ΔCt−2 (Control) + ΔCt−3 (Control)}/3; ΔΔCt (Control) = ΔCt (Control) − mean ΔCt (Control). *p < 0.05, **p < 0.01

To investigate the functionality of LOXL1-AS1 in laryngocarcinoma, its expression in laryngocarcinoma cell lines (SNU-899, SNU-46, AMC-HN-8, Tu-177 and M4E) and human normal pharyngeal epithelial cell line (NP69) was firstly compared. As shown in Fig. 1a, LOXL1-AS1 presented overexpression in laryngocarcinoma cells, and it showed highest level in Tu-177 and M4E cells. Then we inhibited LOXL1-AS1 expression by transfecting three shRNAs targeting LOXL1-AS1 into Tu-177 and M4E cells (Fig. 1b). Next, we detected that the absence of LOXL1-AS1 blocked the proliferation of Tu-177 and M4E cells (Fig. 1c, d). Meanwhile, it was demonstrated that knocking down LOXL1-AS1 obviously impeded the migration of Tu-177 and M4E cells (Fig. 1e, f). Furthermore, we discovered via IF assay that the signals of E-cadherin was elevated while N-cadherin expression declined upon LOXL1-AS1 inhibition (Fig. 1g). Together, LOXL1-AS1 promotes laryngocarcinoma cell growth, migration and EMT.

### LOXL1-AS1 sponges miR-589-5p in laryngocarcinoma cells

**Fig. 2:**
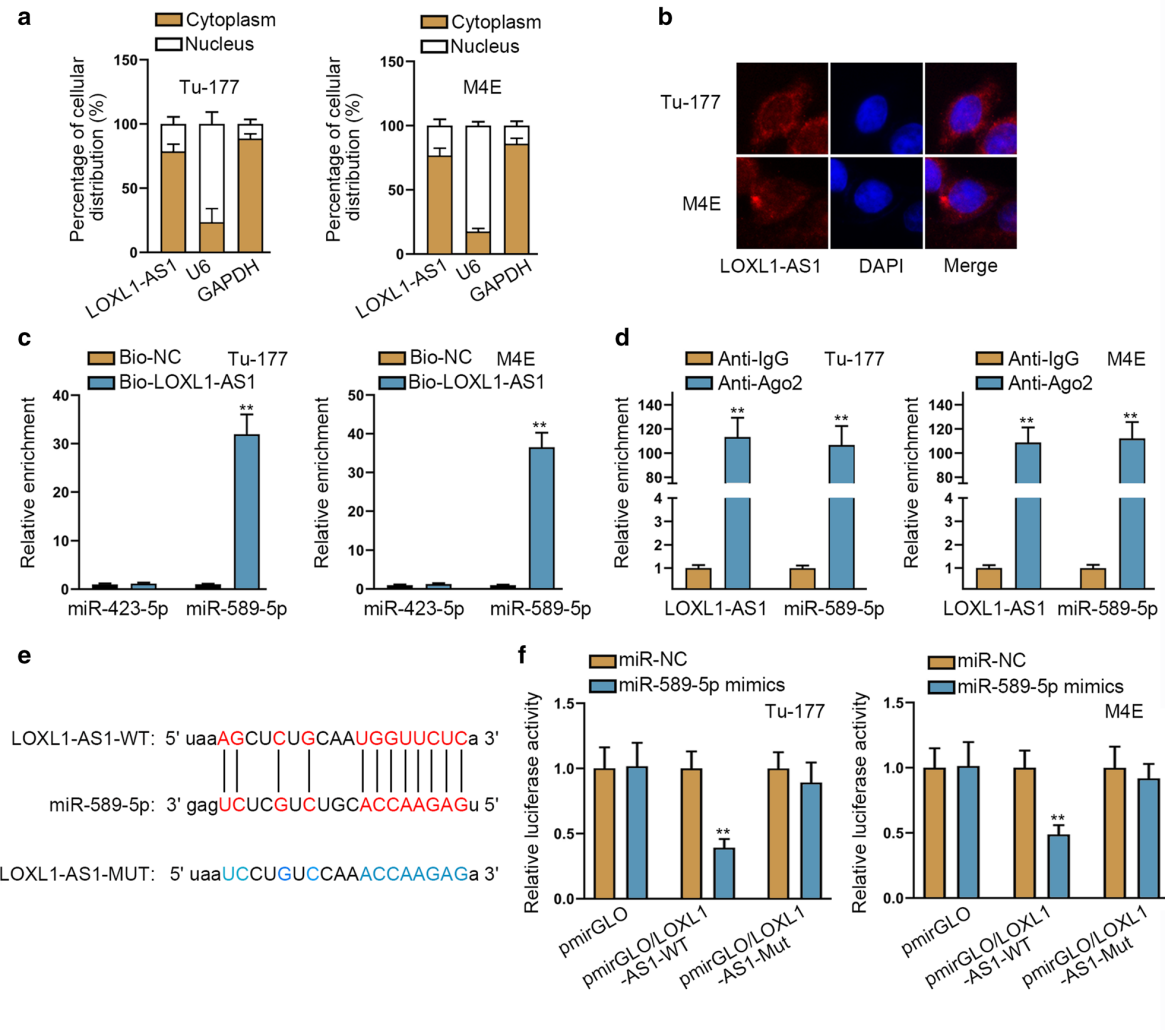
LOXL1-AS1 sponges miR-589-5p in laryngocarcinoma cells. **a**, **b** Nuclear separation and FISH experiments confirmed the main cytoplasmic location of LOXL1-AS1 in both Tu-177 and M4E cells. **c** RNA pull down assay detected that the high enrichment of miR-589-5p but not miR-423-5p in Bio-LOXL1-AS1 groups. **d** Ago2-RIP experiments illustrated the strong enrichment of LOXL1-AS1 and miR-589-5p in anti-Ago2 groups. **e** A binding site of miR-589-5p to LOXL1-AS1 was presented by using starBase. **f** Luciferase reporter assays examined the declined luciferase activity of pmirGLO/LOXL1-AS1-WT but not pmirGLO or pmirGLO/LOXL1-AS1-Mut under the overexpression of miR-589-5p. The standard deviation of control groups in **c**, **d** and **f** was calculated by 2^−ΔΔCt^ method as follow: ΔCt (Control) = ΔCt (target, Control) – ΔCt (reference, Control); mean ΔCt (Control) = {ΔCt−1 (Control) + ΔCt−2 (Control) + ΔCt−3 (Control)}/3; ΔΔCt (Control) = ΔCt (Control)−mean ΔCt (Control). **p < 0.01

To further determine the downstream mechanism of LOXL1-AS1 in laryngocarcinoma cells, nuclear separation and FISH experiments were conducted. As a result, we validated that LOXL1-AS1 principally existed in the cytoplasm (Fig. 2a, b), suggesting LOXL1-AS1 functioned in laryngocarcinoma cells through post-transcriptional control. Also, we proved that Tu-177 and M4E cells transfected with sh/LOXL1-AS1#1 only led to obvious reduction on LOXL1-AS1 signals compared to the control groups, but did not affect the distribution of LOXL1-AS1 (Additional file 1: Figure S1A). Then, by analyzing starBase (http://starbase.sysu.edu.cn), we found 2 miRNAs (miR-423-5p and miR-589-5p) bound to LOXL1-AS1 (screening condition: high stringency in CLIP Data and 4 cancer types in Pan-Cancer). Then, as indicated by the outcomes of RNA pull down assay, miR-589-5p was abundantly detected in Bio-LOXL1-AS1 groups while miR-423-5p was not (Fig. 2c). Moreover, we discovered the strong harvest of both LOXL1-AS1 and miR-589-5p in anti-Ago2 groups in both the two cells (Fig. 2d). Next, we observed a binding site of miR-589-5p to LOXL1-AS1 using starBase, and also doped out the mutant LOXL1-AS1 sequence without such site (Fig. 2e). Furthermore, it manifested that overexpression of miR-589-5p led to a marked decrease of the luciferase activity of pmirGLO/LOXL1-AS1-WT, without apparent impact on that of pmirGLO/LOXL1-AS1-Mut, let alone that of pmirGLO vector (Fig. 2f). Together, LOXL1-AS1 sponges miR-589-5p in laryngocarcinoma cells.

### MiR-589-5p is a tumor-repressor in laryngocarcinoma

**Fig. 3:**
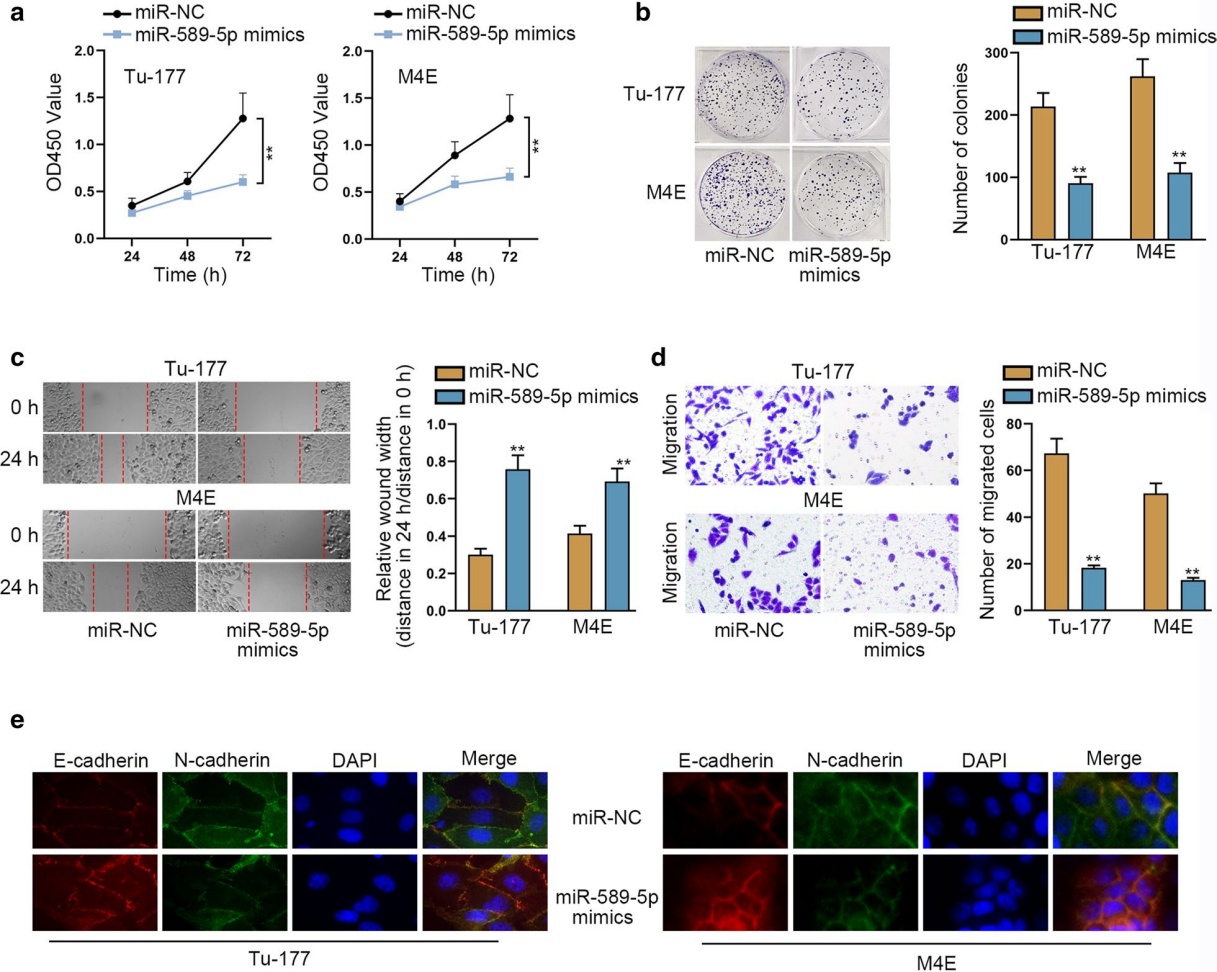
MiR-589-5p restrains cell proliferation, migration and EMT in laryngocarcinoma. **a**, **b** CCK8 and colony formation assays examined the repressive impact of overexpressed miR-589-5p on the proliferation of Tu-177 and M4E cells. **c**, **d**. Wound healing and transwell assays manifested the hampered capacity of migration in Tu-177 and M4E cells in face of up-regulation of miR-589-5p. Relative wound width in **c** was calculated using the relative value of wound widths at 24 h to that at 0 h. **e** IF assessed the augmented expression of E-cadherin and declined level of N-cadherin in Tu-177 and M4E cells with up-regulated miR-589-5p. The standard deviation of control groups in Figure was calculated by 2^−ΔΔCt^ method as follow: ΔCt (Control) = ΔCt (target, Control) – ΔCt (reference, Control); mean ΔCt (Control) = {ΔCt−1 (Control) + ΔCt−2 (Control) + ΔCt−3 (Control)}/3; ΔΔCt (Control) = ΔCt (Control)−mean ΔCt (Control). **p < 0.01

To figure out the role of miR-589-5p in laryngocarcinoma, we assessed the functional changes of laryngocarcinoma cells under miR-589-5p upregulation. It was revealed that overexpressed miR-589-5p suppressed the proliferative capacities of both Tu-177 and M4E cells (Fig. 3a, b). Meanwhile, up-regulation of miR-589-5p also lessened the capacity of migration in Tu-177 and M4E cells (Fig. 3c, d). Furthermore, the expression of E-cadherin was promoted while N-cadherin expression was inhibited in these two laryngocarcinoma cells with elevated miR-589-5p (Fig. 3e). Collectively, miR-589-5p is an inhibitor of laryngocarcinoma progression.

### MiR-589-5p directly targets TRAF6 in laryngocarcinoma cells

**Fig. 4:**
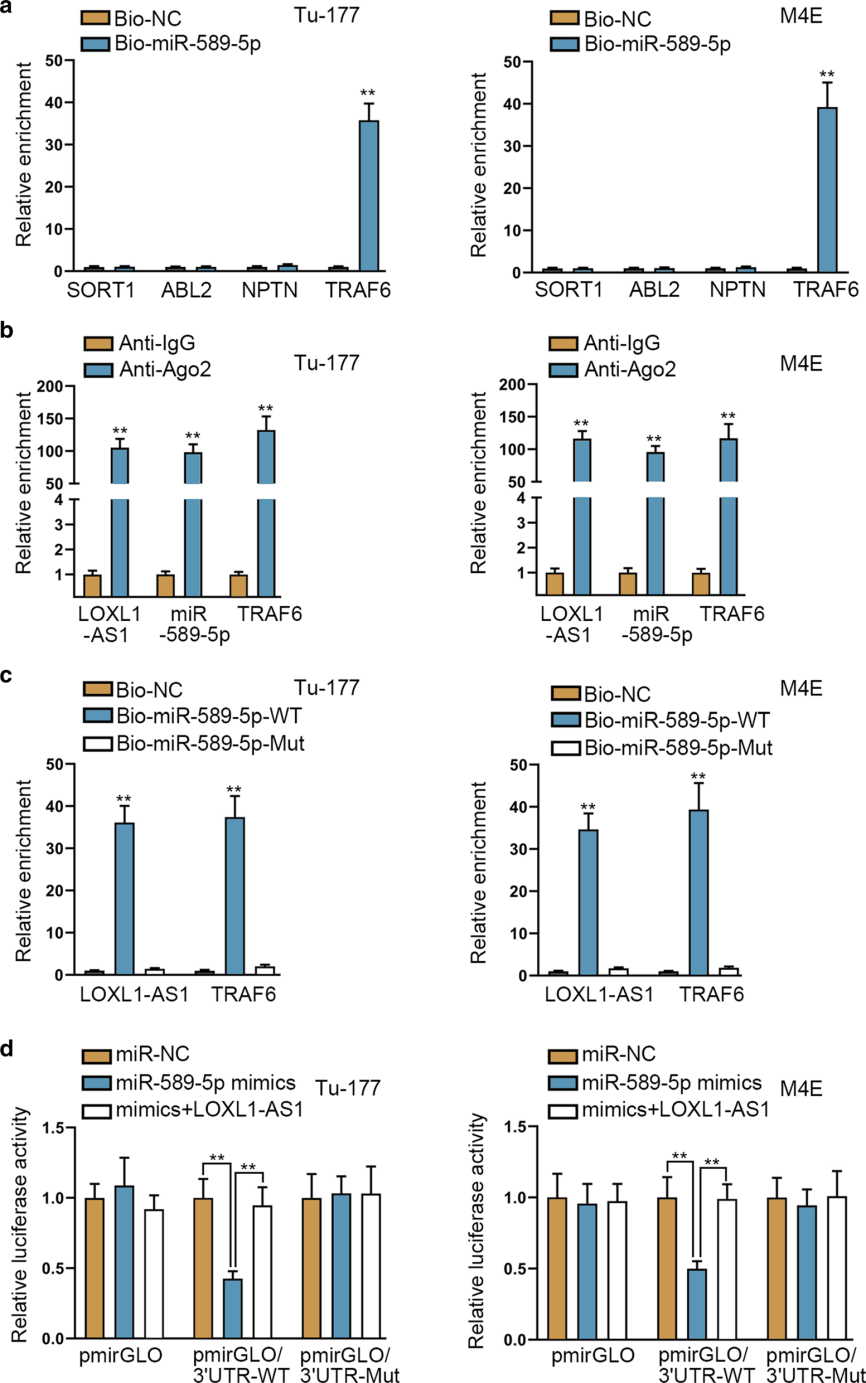
MiR-589-5p directly targets TRAF6 in laryngocarcinoma cells. **a** RIP experiments demonstrated the obvious enrichment of TRAF6 but not SORT1, ABL2 or NPTN in Bio-miR-589-5p groups. **b** Ago2-RIP assays examined that the apparent enrichment of LOXL1-AS1, miR-589-5p and TRAF6 in anti-Ago2 groups. **c** RNA pull down assay disclosed the high enrichment of LOXL1-AS1 and TRAF6 in Bio-miR-589-5p-WT groups. **d** Luciferase reporter assays detected the luciferase activity of pmirGLO/3′UTR-WT/Mut in Tu-177 and M4E cells co-transfected with miR-NC, miR-589-5p mimics, or miR-589-5p mimics + LOXL1-AS1. **p < 0.01

Thereafter, we continued to exploring the downstream mediator of LOXL1-AS1/miR-589-5p axis in laryngocarcinoma. Through bioinformatics programs, we predicted 4 underlying mRNAs binding to miR-589-5p. Subsequently, the results from RIP assays demonstrated that only TRAF6 was significantly enriched in Bio-miR-589-5p groups (Fig. 4a). Further, the outcomes of Ago2-RIP assays disclosed that the enrichment of LOXL1-AS1, miR-589-5p and TRAF6 were obviously detected in anti-Ago2 groups (Fig. 4b). Furthermore, we disclosed that LOXL1-AS1 and TRAF6 could only be captured by Bio-miR-589-5p-WT but never by Bio-miR-589-5p-Mut (Fig. 4c). In addition, the luciferase activity of pmirGLO/3′UTR-WT was apparently reduced in miR-589-5p-enhanced Tu-177 and M4E cells and then reversed by overexpressing LOXL1-AS1. However, no significant change was found in the luciferase activity of pmirGLO/3′UTR-Mut in different groups regardless of transfection (Fig. 4d). Totally, miR-589-5p directly targets TRAF6 in laryngocarcinoma cells.

### LOXL1-AS1 drives malignancy in laryngocarcinoma cells through miR-589-5p/TRAF6 axis

**Fig. 5:**
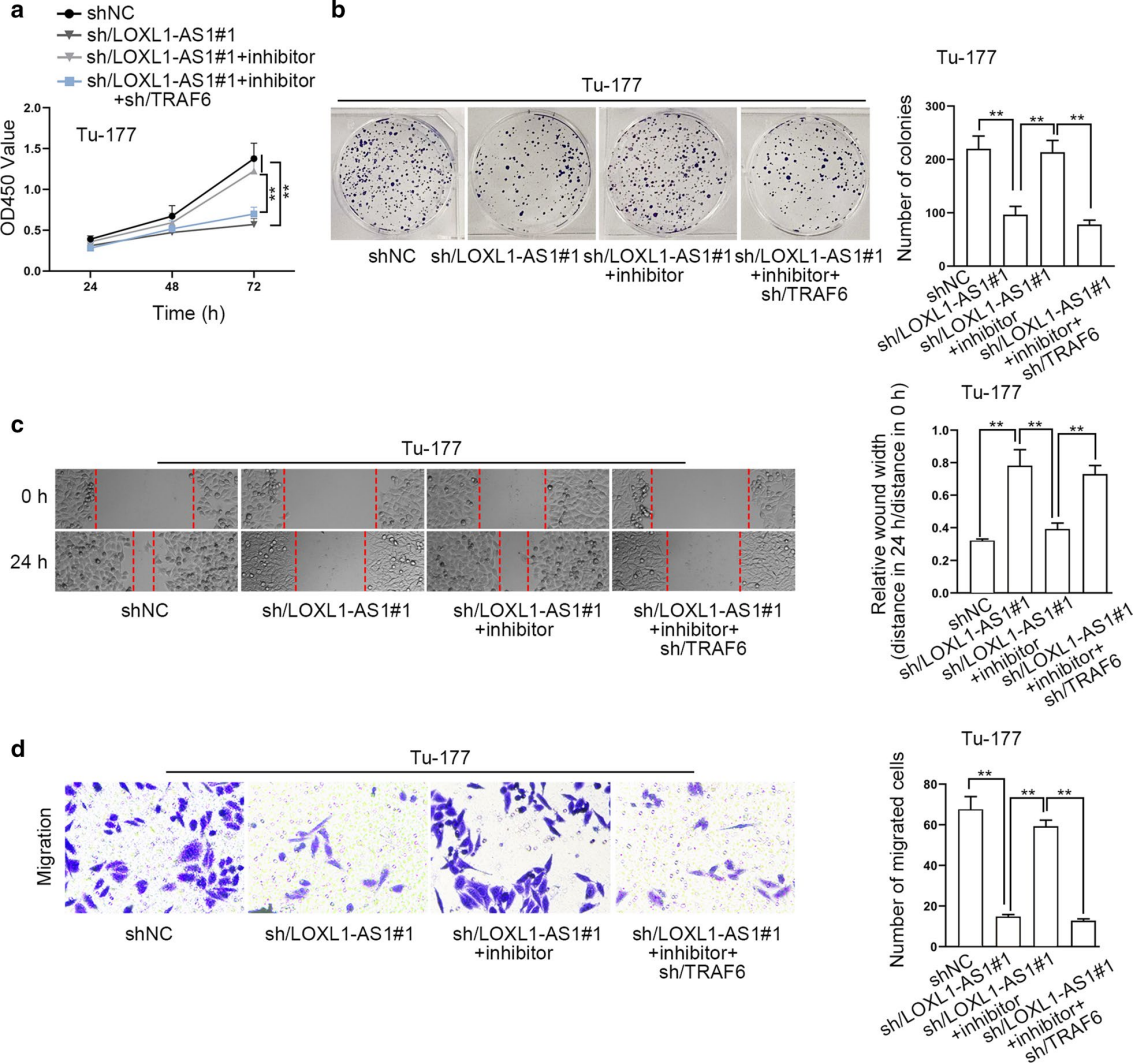
LOXL1-AS1 drives laryngocarcinoma cell proliferation and migration through miR-589-5p/TRAF6 axis. Rescue experiments were conducted in Tu-177 cells transfected with sh-NC, sh/LOXL1-AS1#1, sh/LOXL1-AS1#1 + inhibitor and sh/LOXL1-AS1#1 + inhibitor + sh/TRAF6. **a**, **b** CCK8 and colony formation assays detected the changes on the proliferation of Tu-177 cells in indicated groups. **c**, **d** Wound healing and transwell assays detected the migration ability of indicated Tu-177 cells. Relative wound width in **c** was calculated using the relative value of wound widths at 24 h to that at 0 h. **p < 0.01

To further determine the impact of LOXL1-AS1/miR-589-5p/TRAF6 axis on laryngocarcinoma cells, rescue experiments were conducted by using miR-589-5p inhibitor and shRNAs targeting TRAF6. To test the target efficiency of miR-589-5p inhibitor, we examined its influence on the functional changes induced by miR-589-5p mimics. As a result, we uncovered that the restraining impact of miR-589-5p mimics on the proliferation and migration of laryngocarcinoma cells were fully offset by miR-589-5p inhibitor (Figure S1B-S1E), which confirmed that miR-589-5p inhibitor effectively blocked the function of miR-589-5p in these cells. Furthermore, we also verified that the transfection of shRNAs targeting TARF6 hampered the proliferation and migration of both Tu-177 and M4E cells (Additional file 2: Figure S2A-S2D). Thereafter, we unveiled that silenced LOXL1-AS1-suppressed cell proliferation was then fortified by miR-589-5p inhibition, while such enhancement was further reversed by TRAF6 silencing (Fig. 5a, b). Likewise, the migration ability reduced by LOXL1-AS1 depletion was rescued by miR-589-5p inhibitor, while such rescuing effect was then abolished by down-regulated TRAF6 (Fig. 5c, d). In the meantime, we also implemented in vivo experiments to further test the findings observed in vitro. The results indicated that cells with silenced LOXL1-AS1 grew slower in vivo than controls, and the co-inhibition of miR-589-5p accelerated the in vivo growth, while the accelerating impact was then countervailed by TRFA6 suppression (Additional file 3: Figure S3A-S3B). Totally, LOXL1-AS1 facilitates laryngocarcinoma development through miR-589-5p/TRAF6 signaling.

## Discussion

Recently, increasing literatures have shown the implication of lncRNAs in the biological processes, such as cell proliferation, migration and apoptosis in cancers including laryngocarcinoma. For example, MIR22HG was downregulated in laryngocarcinoma to facilitate malignant behaviors of cells [15]. NEAT1 elevates the capacity of laryngocarcinoma cells to grow and metastasize through modulating miR-29a-3p [16]. Up-regulated TUG1 contributes to tumor growth in laryngocarcinoma via miR-145-5p/ROCK1 pathway [17]. PCAT19 affects laryngocarcinoma cell proliferation by regulating miR-182/PDK4 signaling [18]. In current research, we explored the role of LOXL1-AS1 in laryngocarcinoma cells. As reported previously, LOXL1-AS1 contributes to cell proliferation and metastasis in medulloblastoma through regulating PI3K/AKT signaling [19]. LOXL1-AS1 functions as an oncogene in the progression of osteosarcoma [20]. LOXL1-AS1 contributes to prostate cancer cell growth by regulating miR-541-3p/CCND1 axis [21]. Consistent with the findings of above studies, our study revealed the upregulation of LOXL1-AS1 in laryngocarcinoma cells, and that inhibiting LOXL1-AS1 impeded cell proliferation, migration and EMT in laryngocarcinoma.

Recently, increasing studies have unveiled the strong association between the subcellular localization and the function of lncRNAs [22]. In our work, we confirmed the major distribution of LOXL1-AS1 in laryngocarcinoma cell cytoplasm, while a cytoplasmic lncRNA has a high potential of post-transcriptional regulation [23]. Besides, a ceRNA network, comprised by lncRNA, miRNA and Mrna, is a prevalent manner for the post-transcription control of lncRNA [24]. Moreover, such mechanism has also been reported in laryngocarcinoma [17]. In present study, we unveiled miR-589-5p as the downstream miRNA sponged by LOXL1-AS1 in laryngocarcinoma. MiR-589-5p has been reported as important in suppressing the progression of hepatocellular carcinoma [25], endometrial cancer [26], prostate carcinoma [27], and non-small cell lung cancer [28]. Accordingly, our study also proved that miR-589-5p exerted a inhibitory function in laryngocarcinoma progression.

Subsequently, TRAF6 was confirmed as the downstream of miR-589-5p. In cancer, TRAF6 has been uncovered to work as a tumor-promoter in colorectal cancer [29], oral cancer [30], pancreatic cancer [31], prostate cancer [32] and so on. Similar to these findings, here we also observed that silencing TARF6 resulted in reduced proliferative and migratory capacities of laryngocarcinoma cells. Moreover, we further validated that LOXL1-AS1 was a ceRNA of TRAF6 by absorbing miR-589-5p in laryngocarcinoma cells. Importantly, the final rescue experiments testified that LOXL1-AS1 facilitated in vitro and in vivo tumor growth by targeting miR-589-5p/TRAF6 pathway in laryngocarcinoma. The carcinogenic role of TRAF6 might be attributed to its regulation on NF-kB/c-jun signaling [29]. In addition, TRAF6 is a E3 ubiquitin ligase so that to affect the ubiquitination of diverse proteins, such as p85α [33] and MST1 [31]. Based on these findings, we deduced that the impact of LOXL1-AS1 on laryngocarcinoma development also relied on its regulation on TRAF6, which has powerful functions in above different pathways.

## Conclusion

Our study mainly recognized the promoting influence of LOXL1-AS1/miR-589-5p/TRAF6 signaling on laryngocarcinoma development, which and this might offer a novel biological marker and therapeutic target for the treatment of laryngocarcinoma.

## Supplementary information

**Additional file 1: Figure S1.** A. FISH detected the reduced LOXL1-AS1 signals in Tu-177 and M4E cells after transfected with sh/LOXL1-AS1#1. B-D. Functional assays (CCK-8, colony formation, wound healing and transwell assays) detected the indeed inhibition of miR-589-5p inhibitor on miR-589-5p in Tu-177 and M4E cells. Relative wound width in Figure S1D was calculated using the relative value of wound widths at 24 h to that at 0 h. **p < 0.01.

**Additional file 2: Figure S2.** A, B. The suppressive impact of TRAF6 depletion on the proliferation of Tu-177 and M4E cells was estimated by CCK-8 and colony formation assays. C-D. Wound healing and transwell assays determined the restrained migration of Tu-177 and M4E cells under TRAF6 deficiency. Relative wound width in Figure S2C was calculated using the relative value of wound widths at 24 h to that at 0 h. **p < 0.01.

**Additional file 3: Figure S3.** A. The representative pictures and the corresponding growth curves of tumors originated from Tu-177 cells transfected with sh/NC, sh/LOXL1-AS1#1, sh/LOXL1-AS1#1 + antagomir-589-5p, or sh/LOXL1-AS1#1 + antagomir-589-5p + sh/TRAF6. B. The weight of tumors excised from above four groups. **p < 0.01.

## Data Availability

Research data have been presented within the manuscript and the additional files.

## Abbreviations

lncRNA: Long non-coding RNA
miRNA: MicroRNA
LOXL1-AS1: LOXL1 antisense RNA 1
TRAF6: TNF receptor associated factor 6
ceRNA: Competing endogenous RNA
EMT: Epithelial-mesenchymal transition
FISH: Fluorescence in situ hybridization
CCK8: Cell counting kit-8
RT-qPCR: Quantitative real time polymerase chain reaction
RIP: RNA immunoprecipitation
GAPDH: Glyceraldehyde-3-phosphate dehydrogenase
SD: Standard deviation

## References

[1] Steuer CE, El-Deiry M, Parks JR, Higgins KA, Saba NF (2017). An update on larynx cancer. CA Cancer J Clin.

[2] Zhang SY, Lu ZM, Luo XN, Chen LS, Ge PJ, Song XH, Chen SH, Wu YL (2013). Retrospective analysis of prognostic factors in 205 patients with laryngeal squamous cell carcinoma who underwent surgical treatment. PLoS ONE.

[3] Ma L, Bajic VB, Zhang Z (2013). On the classification of long non-coding RNAs. RNA Biol.

[4] Niu ZS, Niu XJ, Wang WH (2017). Long non-coding RNAs in hepatocellular carcinoma: potential roles and clinical implications. World J Gastroenterol.

[5] Huo X, Han S, Wu G, Latchoumanin O, Zhou G, Hebbard L, George J, Qiao L (2017). Dysregulated long noncoding RNAs (lncRNAs) in hepatocellular carcinoma: implications for tumorigenesis, disease progression, and liver cancer stem cells. Mol Cancer.

[6] Zhou N, Si Z, Li T, Chen G, Zhang Z, Qi H (2016). Long non-coding RNA CCAT2 functions as an oncogene in hepatocellular carcinoma, regulating cellular proliferation, migration and apoptosis. Oncol Lett.

[7] Dong HT, Liu Q, Zhao T, Yao F, Xu Y, Chen B, Wu Y, Zheng X, Jin F, Li J (2020). Long non-coding RNA LOXL1-AS1 drives breast cancer invasion and metastasis by antagonizing miR-708-5p expression and activity. Mol Ther Nucleic Acids.

[8] Wang H, Li L, Yin L (2018). Silencing LncRNA LOXL1-AS1 attenuates mesenchymal characteristics of glioblastoma via NF-κB pathway. Biochem Biophys Res Commun.

[9] Bai T, Liu Y, Li B (2019). LncRNA LOXL1-AS1/miR-let-7a-5p/EGFR-related pathway regulates the doxorubicin resistance of prostate cancer DU-145 cells. IUBMB Life.

[10] Zhang B, Zhou M, Zou L, Miao J, Wang Y, Li Y, Lu S, Yu J (2019). Long non-coding RNA LOXL1-AS1 acts as a ceRNA for miR-324-3p to contribute to cholangiocarcinoma progression via modulation of ATP-binding cassette transporter A1. Biochem Biophys Res Commun.

[11] Zong W, Feng W, Jiang Y, Cao Y, Ke Y, Shi X, Ju S, Cong H, Wang X, Cui M (2020). LncRNA CTC-497E214 promotes the progression of gastric cancer via modulating miR-22/NET1 axis through RhoA signaling pathway. Gastric Cancer.

[12] Li M, Cai O, Tan S (2019). LOXL1-AS1 drives the progression of gastric cancer via regulating miR-142-5p/PIK3CA axis. OncoTargets Ther.

[13] Xie N, Fei X, Liu S, Liao J, Li Y (2019). LncRNA LOXL1-AS1 promotes invasion and proliferation of non-small-cell lung cancer through targeting miR-324-3p. Am J Transl Res.

[14] Yang X, Xing G, Liu S, Li B, He Y, Wang F (2020). LncRNA LOXL1-AS1 promotes endometrial cancer progression by sponging miR-28-5p to upregulate RAP1B expression. Biomed Pharmacother.

[15] Chen H, Ali M, Ruben A, Stelmakh D, Pak M (2020). E2F6-mediated downregulation of MIR22HG facilitates the progression of laryngocarcinoma by targeting the miR-5000-3p/FBXW7 axis. Mol Cell Biol.

[16] Liu T, Wang W, Xu YC, Li ZW, Zhou J (2019). Long noncoding RNA NEAT1 functions as an oncogene in human laryngocarcinoma by targeting miR-29a-3p. Eur Rev Med Pharmacol Sci.

[17] Zhuang S, Liu F, Wu P (2019). Upregulation of long noncoding RNA TUG1 contributes to the development of laryngocarcinoma by targeting miR-145-5p/ROCK1 axis. J Cell Biochem.

[18] Xu S, Guo J, Zhang W (2019). lncRNA PCAT19 promotes the proliferation of laryngocarcinoma cells via modulation of the miR-182/PDK4 axis. J Cell Biochem.

[19] Gao R, Zhang R, Zhang C, Liang Y, Tang W (2018). LncRNA LOXL1-AS1 promotes the proliferation and metastasis of medulloblastoma by activating the PI3K/AKT pathway. Anal Cell Pathol.

[20] Chen S, Li W, Guo A (2019). LOXL1-AS1 predicts poor prognosis and promotes cell proliferation, migration, and invasion in osteosarcoma. Biosci Rep.

[21] Long B, Li N, Xu XX, Li XX, Xu XJ, Liu JY, Wu ZH (2018). Long noncoding RNA LOXL1-AS1 regulates prostate cancer cell proliferation and cell cycle progression through miR-541-3p and CCND1. Biochem Biophys Res Commun.

[22] Chen L-L (2016). Linking long noncoding RNA localization and function. Trends Biochem Sci.

[23] Noh JH, Kim KM, McClusky WG, Abdelmohsen K, Gorospe M (2018). Cytoplasmic functions of long noncoding RNAs. Wiley Interdiscip Rev RNA.

[24] Tay Y, Rinn J, Pandolfi PP (2014). The multilayered complexity of ceRNA crosstalk and competition. Nature.

[25] Zhu Q, Luo Z, Lu G, Gui F, Wu J, Li F, Ni Y (2018). LncRNA FABP5P3/miR-589-5p/ZMYND19 axis contributes to hepatocellular carcinoma cell proliferation, migration and invasion. Biochem Biophys Res Commun.

[26] Wang Y, Dong L, Liu Y (2019). Targeting thyroid receptor interacting protein 6 by microRNA-589-5p inhibits cell proliferation, migration, and invasion in endometrial carcinoma. Cancer Biother Radiopharm.

[27] Ji L, Jiang X, Mao F, Tang Z, Zhong B (2019). miR-589-5p is downregulated in prostate cancer and regulates tumor cell viability and metastasis by targeting CCL-5. Mol Med Rep.

[28] Liu C, Lv D, Li M, Zhang X, Sun G, Bai Y, Chang D (2017). Hypermethylation of miRNA-589 promoter leads to upregulation of HDAC5 which promotes malignancy in non-small cell lung cancer. Int J Oncol.

[29] Zhu G, Cheng Z, Huang Y, Zheng W, Yang S, Lin C, Ye J (2019). TRAF6 promotes the progression and growth of colorectal cancer through nuclear shuttle regulation NF-kB/c-jun signaling pathway. Life Sci.

[30] Wu YH, Wu WS, Lin LC, Liu CS, Ho SY, Wang BJ, Huang BM, Yeh YL, Chiu HW, Yang WL (2018). Bortezomib enhances radiosensitivity in oral cancer through inducing autophagy-mediated TRAF6 oncoprotein degradation. J Exp Clin Cancer Res.

[31] Li JA, Kuang T, Pu N, Fang Y, Han X, Zhang L, Xu X, Wu W, Wang D, Lou W (2019). TRAF6 regulates YAP signaling by promoting the ubiquitination and degradation of MST1 in pancreatic cancer. Clin Exp Med.

[32] Aripaka K, Gudey SK, Zang G, Schmidt A, Åhrling SS, Österman L, Bergh A, von Hofsten J, Landström M (2019). TRAF6 function as a novel co-regulator of Wnt3a target genes in prostate cancer. EBioMedicine.

[33] Hamidi A, Song J, Thakur N, Itoh S, Marcusson A, Bergh A, Heldin CH, Landström M (2017). TGF-β promotes PI3K-AKT signaling and prostate cancer cell migration through the TRAF6-mediated ubiquitylation of p85α. Sci Signal.

