# Supplementary figures and images for: LOXL1-AS1 contributes to the proliferation and migration of laryngocarcinoma cells through miR-589-5p/TRAF6 axis
Source: Cancer Cell Int. 2020 Oct 13;20:504. doi: 10.1186/s12935-020-01565-5 (PMC7552551; doi:10.1186/s12935-020-01565-5)

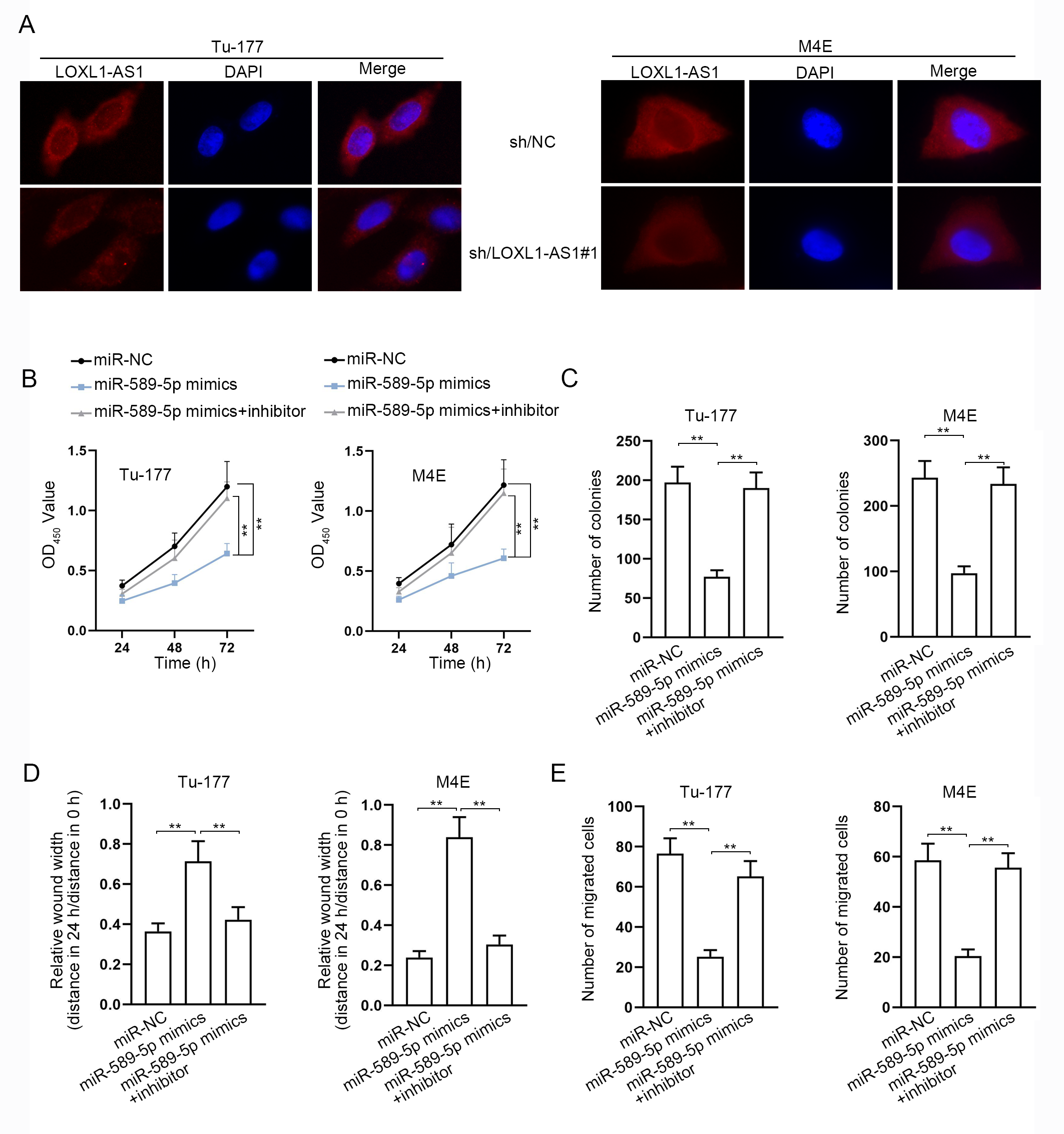

Supplement: Supplementary file 1 — Additional file 1: Figure S1. A. FISH detected the reduced LOXL1-AS1 signals in Tu-177 and M4E cells after transfected with sh/LOXL1-AS1#1. B-D. Functional assays (CCK-8, colony formation, wound healing and transwell assays) detected the indeed inhibition of miR-589-5p inhibitor on miR-589-5p in Tu-177 and M4E cells. Relative wound width in Figure S1D was calculated using the relative value of wound widths at 24 h to that at 0 h. **p < 0.01. [file 12935_2020_1565_MOESM1_ESM.tif]

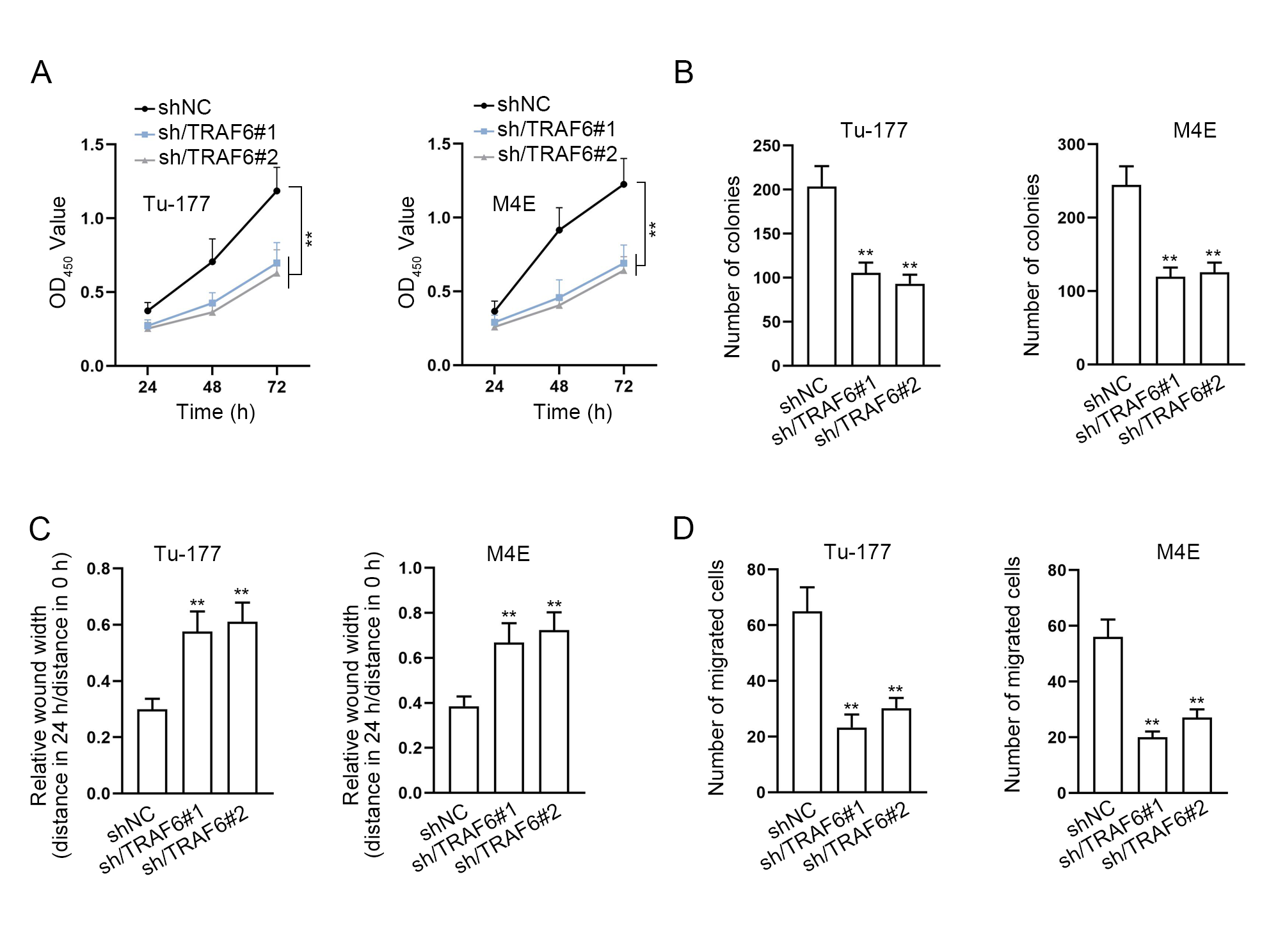

Supplement: Supplementary file 2 — Additional file 2: Figure S2. A, B. The suppressive impact of TRAF6 depletion on the proliferation of Tu-177 and M4E cells was estimated by CCK-8 and colony formation assays. C-D. Wound healing and transwell assays determined the restrained migration of Tu-177 and M4E cells under TRAF6 deficiency. Relative wound width in Figure S2C was calculated using the relative value of wound widths at 24 h to that at 0 h. **p < 0.01. [file 12935_2020_1565_MOESM2_ESM.tif]

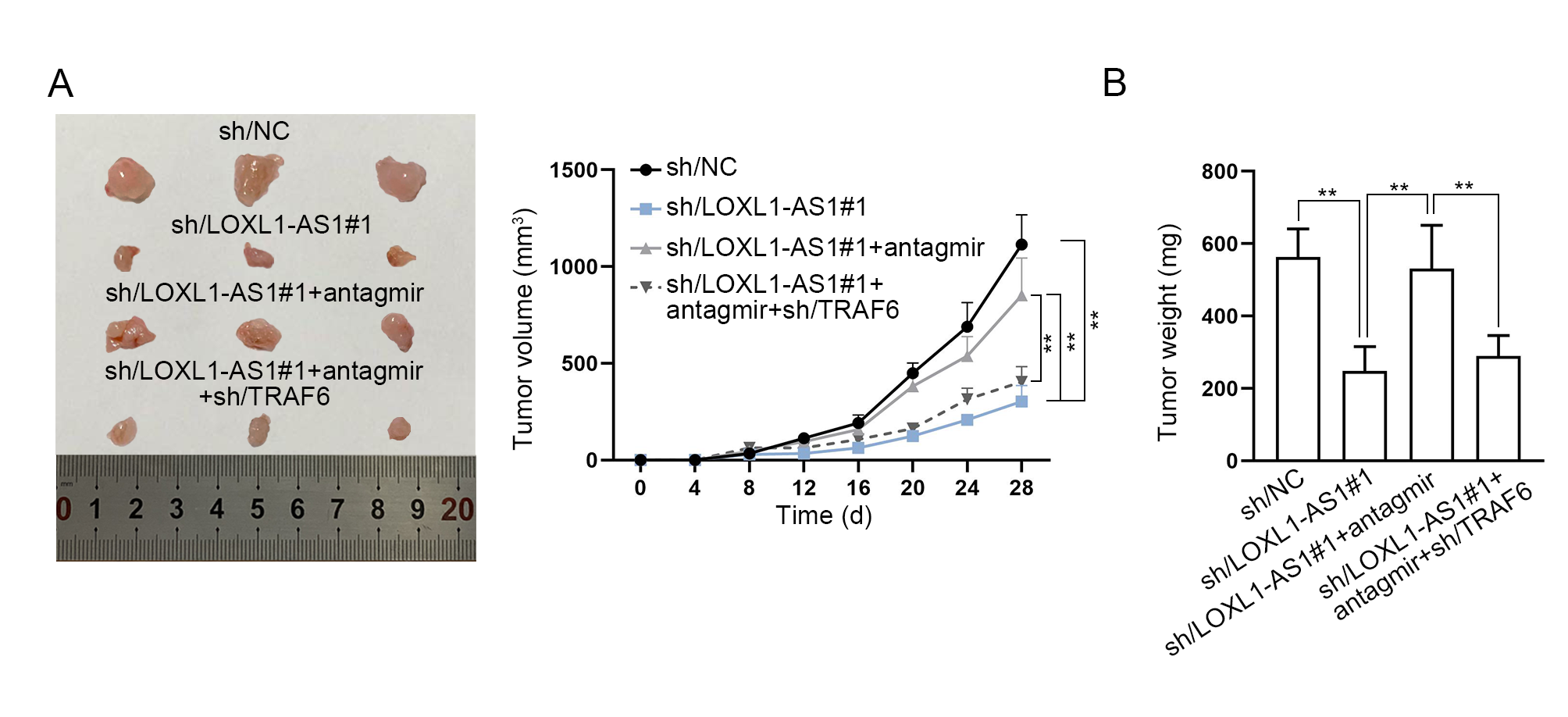

Supplement: Supplementary file 3 — Additional file 3: Figure S3. A. The representative pictures and the corresponding growth curves of tumors originated from Tu-177 cells transfected with sh/NC, sh/LOXL1-AS1#1, sh/LOXL1-AS1#1 + antagomir-589-5p, or sh/LOXL1-AS1#1 + antagomir-589-5p + sh/TRAF6. B. The weight of tumors excised from above four groups. **p < 0.01. [file 12935_2020_1565_MOESM3_ESM.tif]
